# *Trichoderma stromaticum* spores induce autophagy and downregulate inflammatory mediators in human peripheral blood-derived macrophages

**DOI:** 10.1016/j.crmicr.2022.100145

**Published:** 2022-06-18

**Authors:** Lucilla Silva Oliveira-Mendonça, Érica Araújo Mendes, Julyanna Oliveira Castro, Mylene Melo Silva, Andréa Gonçalves Santos, Carla Martins Kaneto, Sandro Oliveira Dias, Ivan Bezerra Allaman, Marcos André Vannier-Santos, Juneo Freitas Silva, Danillo Gardenal Augusto, Danielle Oliveira dos Anjos, Nailma Aprigio Silva Santos, Kamila Pontes Lima, Maria Fátima Horta, George Rego Albuquerque, Márcio Gilberto Cardoso Costa, Izaltina Silva-Jardim, Jane Lima dos Santos

**Affiliations:** a Departamento de Ciências Biológicas, Universidade Estadual de Santa Cruz, Bahia, Brazil; b Departamento de Microbiologia, Instituto de Ciências Biológicas, Universidade de São Paulo, São Paulo, Brazil; c Departamento de Ciências Exatas e Tecnológicas, Universidade Estadual de Santa Cruz, Bahia, Brazil; d Laboratório de Inovações em Terapias, Ensino e Bioprodutos, Instituto Oswaldo Cruz, Fundação Oswaldo Cruz, Rio de Janeiro, RJ, Brazil; e Laboratório de Genética Molecular Humana, Universidade Federal do Paraná, Curitiba, Brazil; f Departamento de Bioquímica e Imunologia, Instituto de Ciências Biológicas, Belo Horizonte, MG, Brazil; g Departamento de Ciências Agrárias e Ambientais, Universidade Estadual de Santa Cruz, Ilhéus, BA, Brazil

**Keywords:** Biofungicide, *Trichoderma*, microRNAs, Autophagy, ROS, Inflammasomes

## Abstract

*Trichoderma* spp. are usually considered safe and normally used as biocontrol and biofertilization. Safety for human health is evaluated by several tests that detect various effects such as allergenicity, toxicity, infectivity, and pathogenicity. However, they do not evaluate the effects of the agent upon the immune system. The aim of this study was to investigate the interaction between *T. stromaticum* spores and mammalian cells to assess the immunomodulatory potential of the spores of this fungus. First, mouse macrophage cell line J774 and human macrophages were exposed to fungal spores and analyzed for structural features, through scanning and transmission electron microscopy. Then, various analysis were performed in human macrophages as to their effect in some functional and molecular aspects of the immune system through immunocytochemistry, flow cytometry and gene expression assays. We demonstrated that *T. stromaticum* spores induces autophagy and autophagy-related genes (ATGs) and downmodulate inflammatory mediators, including ROS, NLRP3, the cytokines IL-1β, IL-18, IL-12 and IL-10, as well as TLR2, TLR4, miR-146b and miR-155, which may lead to an augmented susceptibility to pathogens. Our study shows the extension of damages the biofungicide Tricovab® can cause in the innate immune response. Further studies are necessary to elucidate other innate and adaptive immune responses and, consequently, the safety of this fungus when in contact with humans.

## Highlights

• *T. stromaticum* biocontrol agent induces autophagy, up-regulating autophagy-related genes
• *T. stromaticum* modulates expression of micro RNAs that control imune response
• *T. stromaticum* dow-nregulates expression of TLR2, TLR4, CLEC7A, NLRP3, IL-10, IL1β and IL18
• *T. stromaticum* modulates ROS production

## Graphical Abstract

**Figure:**
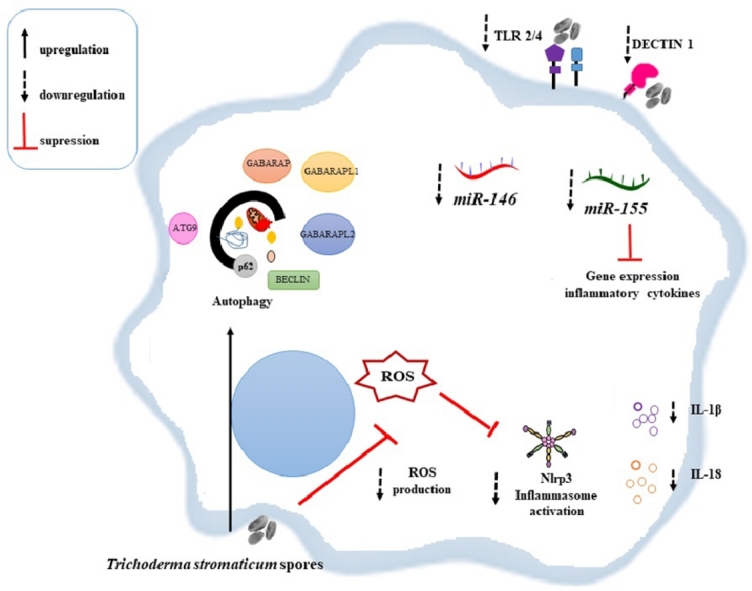

## 1 Introduction

The high demand for food production has elicited an increase use of chemical pesticides for controlling agronomic crop pests (de O. Gomes et al., 2020). However, the indiscriminate use of chemical pesticides may result in contamination of the soil, resistance of weeds and, importantly, human diseases, such as asthma (Henneberger et al., 2014; Hoppin et al., 2009) or other respiratory diseases (Sankoh et al., 2016; Ye et al., 2017) and lung cancer (Alavanja et al., 2004; Bonner et al., 2017), greatly impacting public health (Alori and Babalola, 2018). Therefore, attempts to find safe alternatives to chemicals pesticides drove attention to biological agents that can control microorganisms’ growth with minor environmental burden. One of these agents is the fungus of the genus *Trichoderma*, used in approximately 90% of all documented experimentations for plant diseases management (Adnan et al., 2019). The genus *Trichoderma* represent around 60% of all worldwide used biofungicides (Mukherjee et al., 2012; Thambugala et al., 2020). Specifically, biofungicides containing *T. stromaticum* spores, commercially known as Tricovab®, are used in Brazil and other countries for controlling the witches’ broom disease of cocoa trees caused by the phytopathogen *Moniliophthora perniciosa* ( del Carmen et al., 2021, Medeiros et al., 2010).

Despite their benefits for cocoa production, the spores can survive and germinate in the environment with a high probability of being inhaled or swallowed by humans or other animals (Hansen et al., 2010; Luangsa-Ard et al., 2011). The use of spores may therefore affect particularly, but not only, immunosuppressed individuals chronically exposed (Konstantinovas et al., 2017). Nevertheless, how *Trichoderma* spores may affect innate or adaptive responses is poorly understood.

Previous results from our group have demonstrated that, when inhaled by C57BL/6 mice, *T. stromaticum* spores causes a discrete cell infiltrate in the lungs. Inhalation of spores also causes a reduction in the production of interleukin (IL)-10 in the bronchoalveolar lavage, as well as an inhibition of IL-10 and IFN-γ production in ConA-stimulated spleen cells. We have also shown that, in vitro, spores impair splenocyte proliferation, as well as the production of reactive oxygen species (ROS) and nitric oxide (NO) in neutrophils and macrophages, respectively. In addition, spores inhibited the expression of the Pattern Recognition Receptors (PRRs) dectin-1, TLR2 and TLR4 in both cell types (Alves-Filho et al., 2011). These findings showed that the *T. stromaticum* biopesticide may exert immunosuppressive effects in vitro and in vivo. Similarly, we have also shown that macrophages from mice intraperitoneally exposed to *T. asperelloides* spores have dectin-1 and TLR2, as well as the number of cells and their ability to internalize *Candida parapsilosis* ex vivo reduced (dos Santos et al., 2017).

TLRs play a pivotal role in the innate immunity and may be regulated by microRNAs (Chen et al., 2013; Quinn and O'Neill, 2011). TLRs, in particular TLR2 and TLR4, activate the transcription factor NF-κB, which induces transcription of several genes, including those of the pro-inflammatory cytokines IL-1β, IL-6, IL-12, TNF-α (Cohen, 2017; Fitzgerald and Kagan, 2020; Nahid et al., 2011). It has been shown that the microRNAs miR-146 and miR-155, expressed in macrophages and monocytes, affects innate and adaptive immunity in response to microbial infection, mainly after lipopolysaccharide (LPS) stimulation (Maudet et al., 2014). When overexpressed, miR-146 downregulates the innate immune response mediated by TLR2 (Lee et al., 2016; Nahid et al., 2009). While miR-146a is associated to an anti-inflammatory response and macrophage polarization to a M2 profile, miR155 induces the M1 profile and upregulation of an inflammatory immune response (Essandoh et al., 2016; Pourgholi et al., 2017). It has been demonstrated that inhibition of *TLR2* increases the expression of miR-146 and, consequently, leads to a downregulation of pro-inflammatory signaling molecules, such as IL-1β (Griss et al., 2016).

Autophagy is the process by which dysfunctional components are intracellularly degraded by lysosomes, a cell process to recycle organelles, and to eliminate intracellular microorganisms (Chun and Kim, 2018). This is a highly conserved mechanism in which poorly folded proteins and damaged organelles, among other elements, are encompassed by double membranes. This process generates autophagosomes, which are degraded by lysosomal enzymes, mediated by a central set of proteins - autophagy-related proteins (ATG) and other components of the autophagy interaction network (Levine and Kroemer, 2019; Tang et al., 2012). In mice and humans, autophagy regulates several innate immunity responses, such as monocyte survival and differentiation, LC3-associated phagocytosis (LAP), and activation of the NOD-like receptors family of pyrin domain-containing-3 (NLRP3), as well as antigen presentation and production of cytokines (Germic et al., 2019). On the other hand, autophagy is associated with the restoration of intracellular homeostasis after excessive activation of innate and adaptive responses by regulating the immune tolerance process (Wu et al., 2019). Changes in PRRs, including TLRs and NLRs, can interfere with cascades of immune cells activation, including autophagy and represents a form of survival and one of the first host defense strategies (Tang et al., 2012).

Since macrophages are key cells for the elimination of various microorganisms (Chua et al., 2021; Piacenza et al., 2019; Weiss and Schaible, 2015) and since our previous data using a murine model indicated that the presence of *T. stromaticum* spores negatively impact some murine inflammatory innate responses (Alves-Filho et al., 2011; dos Santos et al., 2017) we decided, in the present work, to switch to a more relevant in vitro human model to carry out a more comprehensive study. We investigated here whether some of the effects we demonstrated in the murine immune cells also occurred in the human model. We are considering the hypothesis that the negative regulation is associated with the regulation of autophagy. For this, we investigated the occurrence of autophagy and the autophagy-related genes (ATGs) in human macrophages, after contact with *T. stromaticum* spores. In doing so, we also took the chance to investigate other functional alterations on macrophage responses and their possible association with autophagy. These include the expression of NLPR3, besides the PRRs dectin-1, TLR2, TLR4, and micro RNAs, important for the regulation of immune responses, as well as the production of ROS and various cytokines, which could possibly account for an immunosuppressive outcome.

## 2 Material and Methods

### 2.1 Fungal growth and spore collection

*T. stromaticum* spores were isolated from the commercially available biofungicide Tricovab® and cultured on potato dextrose agar (PDA) plates. The cultures were maintained at 26℃ for 10 days. Subsequently, spores were obtained from cultures by washing the plate with sterile distilled water, followed by centrifugation at 1160 x g for 5 minutes. Pellets were washed three times in sterile distilled water and resuspended in phosphate-buffered saline (PBS). The spores were then counted on a hemocytometer.

### 2.2 Human subjects

Peripheral blood was collected from 12 healthy volunteers from Ilhéus city, Bahia, Brazil (56% man and 44% female) with age ranging from 20-44 years and not exposed to occupational activities related to the studied fungus. We excluded individuals with reported health medical conditions or using medication. All subjects voluntarily agreed to participate and signed an informed consent before their inclusion in this study. The study was conducted in accordance with the Declaration of Helsinki and Brazilian Federal laws and approved by State University of Santa Cruz's Ethics Committee (Project identification code 550.382/2014).

### 2.3 Cell isolation, culture, and microscopy observation

Peripheral blood mononuclear cells (PBMC) were obtained by density barrier method using Histopaque 1077 g/mL (Sigma-Aldrich, Saint Louis, MO), as previously described (Johnston et al., 2015). The cells were washed three times in PBS and resuspended in RPMI 1640 medium (Gibco BRL, Gaithersburg, MD) supplemented with 10% fetal bovine serum (Gibco BRL, Gaithersburg, MD) and 1% antibiotic (penicillin/streptomycin). The cells were counted on a hemocytometer and cell viability were determined by Trypan blue exclusion test (Sigma-Aldrich, Saint Louis, MO) as well as spreading pattern observation.

Human macrophages were obtained by incubation of isolated PBMCs at 37℃ under 5% CO_2_ atmosphere, for 7 days, with fresh medium change every 2 days, according to a previously described methodology (Rios et al., 2017).

Human macrophages were cultured in plates containing or not *T. stromaticum* spores (10:1 cells/spores ratio), at 37℃ and 5% CO_2_ for different period of times (30 min, 3 h, 6 h, and 12 h). Cells were then fixed in 2.5% glutaraldehyde (Sigma-Aldrich, Saint Louis, MO) diluted in 0.1 M sodium cacodylate buffer for 1 hour at 25℃ and post-fixed in 1% osmium tetroxide (Polysciences, Warrington, PA). Fixed cells were dehydrated in ethanol series, dried by the critical point method in a Balzer's apparatus, mounted on stubs, and covered with a 20 nm-thick gold layer. Specimens were observed in a JEOL 5310 scanning electron microscope. Alternatively, cells were stained by May-Grünwald-Giemsa technique (MGG) (Lombard et al., 1994).

### 2.4 Macrophages viability post-exposure to *T. stromaticum* spores

Macrophages were cultured overnight (10^5^ cells in 200 uL of RPMI medium per well) in 96-well plates with or without increasing amounts of *T. stromaticum* spores (10^4^, 10^5^ and 10^6^ spores/well) at 37℃ and 5% CO_2_. Cell viability was verified by the established bromide 3-(4,5-dimethyltiazol-2-yl)-2,5-diphenyl tetrazolium (MTT) assay (Mosmann, 1983) as previously reported (Alves-Filho et al., 2011)

### 2.5 Monodansylcadaverine (MDC) labeling

Cell monolayers were washed and suspended in PBS pH 7.4. 0.05 mM Monodansylcadaverine MDC (Sigma-Aldrich, Saint Louis, MO) was added to the culture, which was incubated at 37℃ for 30 minutes for autophagy labeling. J774 cells were visualized using a fluorescence microscope (Nikon, Japan) at 360-380 nm of excitation wavelength, 525 nm of emission wavelength and images acquired using a digital camera (Coolpix 990, Nikon). A total of 100 fields randomly selected were counted to measure the autophagy induction (Klionsky et al., 2021).

### 2.6 Imunocytochemistry

Microtubule-associated protein light chain 3 (LC3B) is an autophagosome membrane marker so we employed immunocytochemistry with anti-LC3B to evaluate the occurrence of autophagy, on PBMC (Klionsky et al., 2021). Calls were fixed with paraformaldehyde 4% for 20min at 8°C and permeabilized. Cells were subsequently stained with anti-LC3B (ab51520 Abcam, EUA) and the secondary rat anti-IgG labeled with Alexa Fluor® 488 (Invitrogen, EUA), and were observed under the fluorescence microscope. Images were acquired using a digital camera (Coolpix 990, Nikon). Results were represented after counting at least 100 cells in each experimental time point using the software ImageJ®.

### 2.7 Detection of Reactive Oxygen Species

To detect reactive oxygen species (ROS) in macrophages exposed to *T. stromaticum* spores for 30min, 3h, 6, 24h, the fluorescent probe dihydroethidium (DHE Molecular Probes®), was used. For the assay positive control, cells were treated with 100nM Phorbol-12-Myristate-13-Acetat (PMA). Briefly, cells were washed with PBS plus 1% bovine serum albumin (BSA), adjusted to one million, and incubated in the dark at 4°C for 30min in the presence of 50nM DHE. Twenty thousand events were captured using the flow cytometer CytoFLEX S – (Beckman Coulter). Data analysis was made using the software Infinicyt V1.7 (Infinicyt, Cytognos; Salamanca, Spain), and represented as percentage of DHE-stained positive cells (gate A) from three experimental replicates. Controls of fluorescence [Fluorescence-minus-one (FMO)] were used to distinguish the fluorescence of positive cells, as well as a negative control without spores and any other stimulus for threshold definition.

### 2.8 RNA Isolation and cDNA synthesis

Total RNA was isolated using TRIzol (Invitrogen, Walthan, MA), according to the manufacturer's instructions. For each RNA extraction, 250μL of 5x10^6^ once-thawed macrophages/well were added to 750 μL TRIzol LS. were added, and samples were vortexed vigorously for 30 seconds and then allowed to sit at room temperature for 5 minutes. Phase separation was achieved by centrifuging the sample at 12,000 g for 20 minutes at 4℃. After centrifugation, 400 μL of the aqueous phase was carefully transferred to a new tube. RNA was eluted in nuclease-free water by passing a few times through a pipette tip. RNA quality and quantity were measured by using Nanodrop spectrophotometer (ND-1000, Nanodrop Technologies). For miRNA expression analysis, total RNA was used for cDNA synthesis utilizing TaqMan® MicroRNA Reverse Transcription Kit (Thermo Fisher Scientific, Foster City, CA) with Multiscribe Reverse Transcriptase, 10 X Reverse transcription buffer and RNase inhibitor. The mixture was incubated for 30 min at 16 °C, 30 min at 42°C and for 5 min at 85 °C to inactivate the reverse transcriptase and placed on ice. This cDNA was diluted in RNase-free water (15 μl of cDNA obtained above mixed with 45 μl of water) for Real-Time PCR reactions. For gene expression analysis, total RNA was reverse transcribed using the High Capacity cDNA Reverse Transcription Kit (Thermo Fisher Scientific Baltics, Lithuania), following manufacturer's recommendations.

### 2.9 Gene expression analysis

Quantitative PCR amplification was performed with 20 ng of template cDNA, 2X Power SYBR Green Master mix buffer (10 µL) (Thermo Fisher Scientific, Foster City, CA, USA) and 400 to 600 nM forward and reverse primers in a final volume of 20 µL. All reactions were duplicated using QuantStudio 3 Real Time System (Thermo Fisher Scientific, Foster City, CA, USA), under the following conditions: 95℃ for 10 min, followed by 40 cycles at 95℃ for 15 s, and 60℃ for 1 min. The housekeeping gene *GAPDH* was used for normalization. Experiments with coefficients of variation greater than 5% were excluded. For all reactions, each run was completed with a melting curve analysis to the specificity of amplification and lack of primer dimers. Reactions were run in duplicate and a NTC and no-RT were also included. The relative quantification of gene expression was carried out using the 2-ΔΔCT formula and presented as fold change. Table 1 shows the description of the primers used for each reaction.

**TABLE 1:** Primers for the specific genes used in the qPCR analysis.

Gene	Primer
*CLEC7A*	Forward: 5′-GACTGAGGTACCATGGCTCTG-3′ Reverse: 5′-GGAGATGGGTTTTCTTGGGTAGC-3′
*TLR 2*	Forward: 5′-CGGAGAGACTTTGCTCACTC-3′ Reverse: 5′-CGTGTGCTGGATAAGTTCAA-3′
*TLR4*	Forward: 5′-CCGATTCCATTGCTTCTTGC-3′ Reverse: 5′-AGCTCAGGTCCAGGTTCTTGG-3′
*TNF*	Forward: 5′-CAG GCA GTC AGA TCA TCT TC-3′ Reverse: 5′-CTT GAG GGT TTG CTA CAA CA-3′
*IL-12*	Forward: 5′-TGGAGTGCCAGGAGGACAGT-3′ Reverse: 5′-TCTTGGGTGGGTCAGGTTTG-3′
*IL-10*	Forward: 5′-GTGATGCCCCAAGCTGAGA-3′ Reverse: 5′-CACGGCCTTGCTCTTGTTTT-3′
*BECLIN 1*	Forward: 5’ - CAAGATCCTGGACCGTGTCA - 3’ Reverse: 5’ - TGGCACTTTCTGTGGACATCA - 3’
*ATG9*	Forward: 5’ - CTCATCGGGGAGATCTTTGA - 3’ Reverse: 5’ - GACTTGAGCAGGCAAAAAGG - 3’
*SQSTM1*	Forward: 5’ – AAGAACGTTGGGGAGAGTGTG - 3’ Reverse: 5’ - GACTCCAAGGCGATCTTCCTC - 3’
*GABARAP*	Forward: 5’ - ATGTCATTCCACCCACCAGT - 3’ Reverse: 5’ - CAGCAGCTTCACAGACCGTA - 3’
*GABARAPL1*	Forward: 5’ - ATCCGGAAGAGAATCCACCT - 3’ Reverse: 5’ - TCTTCCTCATGATTGTCCTCA - 3’
*GABARAPL2*	Forward: 5’ – AAATATCCCGACAGGGTTCC - 3’ Reverse: 5’ – CAGGAAGATCGCCTTTTCAG – 3’
*NLRP3*	Forward: 5’ - GCAAAAAGAGATGAGCCGAAGT-3′ Reverse: 5’ - GCTGTCTTCCTGGCATATCACA-3′
*IL-18*	Forward: 5’ - GCTGCT GAACCAGTAGAAGAC - 3′ Reverse: 5’ - CCGATTTCCTTGGTCAATGAAGA - 3′
*IL1-β*	Forward: 5’ – CACGATGCACCTGTACGATCA - 3’ Reverse: 5’ – GTTGCTCCATATCCTGTCCCT - 3′
*GAPDH*	Forward: 5′-ATCCCATCACCATCTTCCAG-3′ Reverse: 5′-TGGACTCCACGACGTACTCA-3′

### 2.10 miR-146a/b and miR-155 Expression Analysis

To evaluate the levels of miRNAs, we used the Taqman MicroRNA kit (Thermo Fisher Scientific Foster City, USA). Quantitative PCR amplification mixes contained 20 ηg of cDNA template, Taqman master mix (10 μL) and probes for miR-155 (Cat. # 002571), miR-146a (Cat. #000468) and miR-146b (Cat. # 001097) in a 20 μL final volume (Thermo Fisher Scientific, Foster City, CA). Assays were performed in duplicates, on an ABI7500 real-time PCR system (Thermo Fisher Scientific, Foster City, USA) under standard thermal cycling conditions. The mean cycle threshold (Ct) values from duplicate measurements were used to calculate expression of the target gene, with normalization to an internal control miR-320a (Cat. # 002277) using the 2^-ΔΔCT^. A test was designed to select and validate the reference miRNAs with greater accuracy. The expression of 3 miRNAs as internal controls (U6sn RNA, miR-328-3p, miR-320a) was detected in the samples of each experimental group and their stability was analyzed using the software NormFinder (Andersen et al., 2004). NormFinder is a Microsoft Excel-based application that uses a model-based approach to assign a stability value to each candidate normalizer, accounting for intra- and inter-group variation, with lower stability values indicating higher stability of the candidate. For analysis, the whole data set of input RNA and inter-run calibrator-normalized Ct-values were analyzed with the NormFinder software to calculate stability values derived from the intra and inter-group variability. Analysis revealed that among the 3 candidates, miR-320a was the most stable gene (stability value = 0.263) and suitable as an internal control.Experiments with coefficients of variation greater than 5% were excluded. A no-template control (NTC) and no reverse transcription controls (no-RT) were also included.

### 2.11 Statistical Analysis

In all experiments, a randomized block design with 3 replicates per treatment was used. The analysis of variance (Anova) was used in all experiments, and when the assumptions were violated, the logarithmic transformation was used. In the case of quantitative treatments, when significant differences were detected in Anova, logistic regression was used to adjust the data. As for the MTT assay, Dunett's contrast and student's t-test were used. The level of significance was 5%. Analyses were performed with the R software 3.3.2 version and with the aid of the package Stats 3.3.2 version and Prism software (v. 8, GraphPad). Spearman correlation analysis tests were applied using R (for the correlation matrix).

## 3 Results

### 3.1 Internalization of *T. stromaticum* spores by murine macrophages

**Fig. 1:**
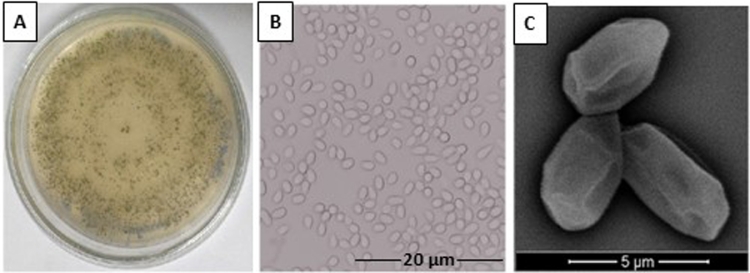
Morphological characteristics of T. stromaticum. (A) Macroscopic appearance of spores in culture; (B) Optical microscopy – Magnification 1000x, (C) Spores accessed by scanning EM displaying surface edges, mainly longitudinally oriented.

Initially, we confirmed that *T. stromaticum* spores isolated from the biofungicide Tricovab® and grown on PDA, exhibited the morphological characteristics expected for the species. We observed that, in 7-days the culture displayed the typical macroscopical dark green color and ruffled surface (Fig. 1A) and microscopical oval-shaped appearance with surface folding (Figs. 1 B and C), as previously reported (De Melo and Faull, 2004).

**Fig. 2:**
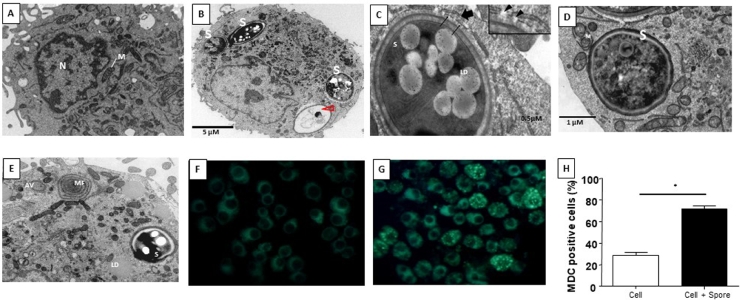
Effects of interaction between *T. stromaticum* spores and J774 cells analyzed with TEM. (**A**) untreated J774 cells showing their typical ultrastructure; (**B - D**) J774 cells exposed to the fungal spores for 24h showing that spores (S) were completely phagocytosed; (**B**) Note the presence of spores with electron-lucent or washed-out cytoplasm, (arrowhead); (**C**) spores were surrounded by membranes (thin arrows) and endoplasmic reticulum cisternae (thick arrow), which were eventually discontinued (inset, arrowheads); (**D**) some spores were completely free within the phagocyte cytoplasm; (**E**) of Myelin-like figures (MF) and autophagosomes (AF- in the cytoplasm of the phagocytes after *T. stromaticum* spore exposure during 24h; Autophagy triggering was analyzed by MDC staining (**F and G**). Control cells not exposed to *T. stromaticum* (F) presented faint and diffuse staining whereas macrophages exposed to spores (G) displayed intense punctate labelling, which was significantly (* = p<0.05) more frequent than in controls (**H**). The bars represent the mean ± SD of three independent experiments. *S - T. stromaticum spores; LD - Lipid Droplets; M – Mitochondria; N – nucleus*

To certify that J774 cells (Fig. 2A) interacted with and internalized *T. stromaticum* spores (Figs 2B-D), we exposed the phagocytic cells to *T. stromaticum* spores. After 24h of culture, we observed by transmission electron microscopy (TEM) that spores were internalized by the phagocytes (Fig. 2B-D), and displayed different sizes and shapes varying from oval to spherical. Most of the internalized spores exhibited electron-dense cytoplasm, suggestive of maturity and viability. Electron-lucent or washed-out cytoplasm were also observed in some of the spores, which suggests cytoplasmic degradation (Fig. 2B - arrowhead). Spores were generally surrounded by a membrane, presumably remnants from the phagocytic vacuole membrane, degraded after ingestion (Fig. 2C, thin arrows and inset) or the endoplasmic reticulum (ER) cistern (Fig. 2C, thick arrows and inset). Eventually spores were found completely free in the phagocyte cytoplasm (Fig. 2D).

This internalization experiment was important to observe that J774 cells that had phagocytosed *T. stromaticum* spores exhibited autophagosomes (AF) and myelin-like figures (MF) and have increased lipid droplets (LD) number (Fig. 2E) when compared to control, spore-free cells (Fig. 2A). Autophagy in cells that had internalized the spores was confirmed by the selective autophagosome marker MDC (Biederbick et al., 1995) that abundantly and intensely labeled the spore-containing cells (Fig. 2G), as opposed to non-exposed cells (Fig. 2F). Quantification of MDC-stained cells showed that J774 cells exposed to the fungus displayed nearly three-fold higher autophagosome formation (72%) than non-exposed controls (25%) (Fig. 2H)

### 3.2 *T. stromaticum* spores are phagocytosed by human macrophage and are not cytotoxic

**Fig. 3:**
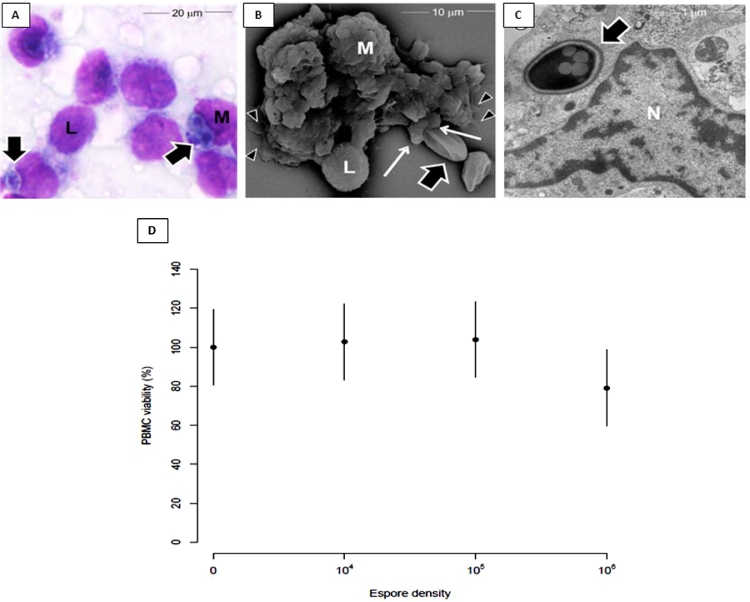
Phagocytic and viability of macrophages exposed to *T. stromaticum* spores. Macrophages exposed to fungal spores for 24h showing that cells (M) ingested spores (thick arrows); (**A**) Light microscopy- note that some macrophages were also attached to lymphocytes (L); (**B**) SEM shows a monocyte tightly adhered to a lymphocyte and phagocytosing *T. stromaticum* spore. Note that monocyte displays pseudopodia both in the phagocytic cup, ingesting the spores (thin arrows) and attaching to the substrate (arrowheads); (**C**) TEM shows that *T. stromaticum* spores were internalized by macrophage after 12h. (D) Determination of cell viability (10^5^ cells/well) after interaction for 18 hours at different spore densities (0, 10^4^, 10^5^ or 10^6^/well) using the MTT assay. The results are presented as mean of three independent experiments in triplicate with confidence interval of 95%;

To verify that, *T. stromaticum* spores interacts and are also phagocytosed by human macrophages, we performed either optical or electron microscopy. Images show that spores were also found inside the human cells, as suggested by light microscopy (Fig. 3A). By scanning electron microscopy (SEM) we noticed that in 30 minutes of interaction, *T. stromaticum* spores could be found free or attached to the cell membrane, showing the beginning of the phagocytic process (Fig. 3B). Macrophages already presented microvillus projections, cell surface ruffles, blebs, and pseudopodia emission in close apposition to adherent fungal spores as well as mediating substrate attachment (Fig. 3B). TEM certified that, in fact, after 12h, spores of *T. stromaticum* were internalized by macrophages (Fig. 3C). The exposure of macrophages to 10^4^, 10^5^, or 10^6^ *T. stromaticum* spores did not affect their viability, as shown by MTT (Fig. 3D).

### 3.3 T. stromaticum spores induce autophagy in human macrophages, which correlates with upregulation of ATG mRNA

Autophagy is an important mechanism of innate host defense against microrganisms and is associated with re-establishment of homeostasis and is even implicated in immune tolerance (Krakauer, 2019; Tian et al., 2019; Wu et al., 2019). Considering our previous results showing a negative regulation of inflammatory mediators in murine cells, we hypothesized that this negative regulation could be associated with autophagy in macrophages.

**Fig. 4:**
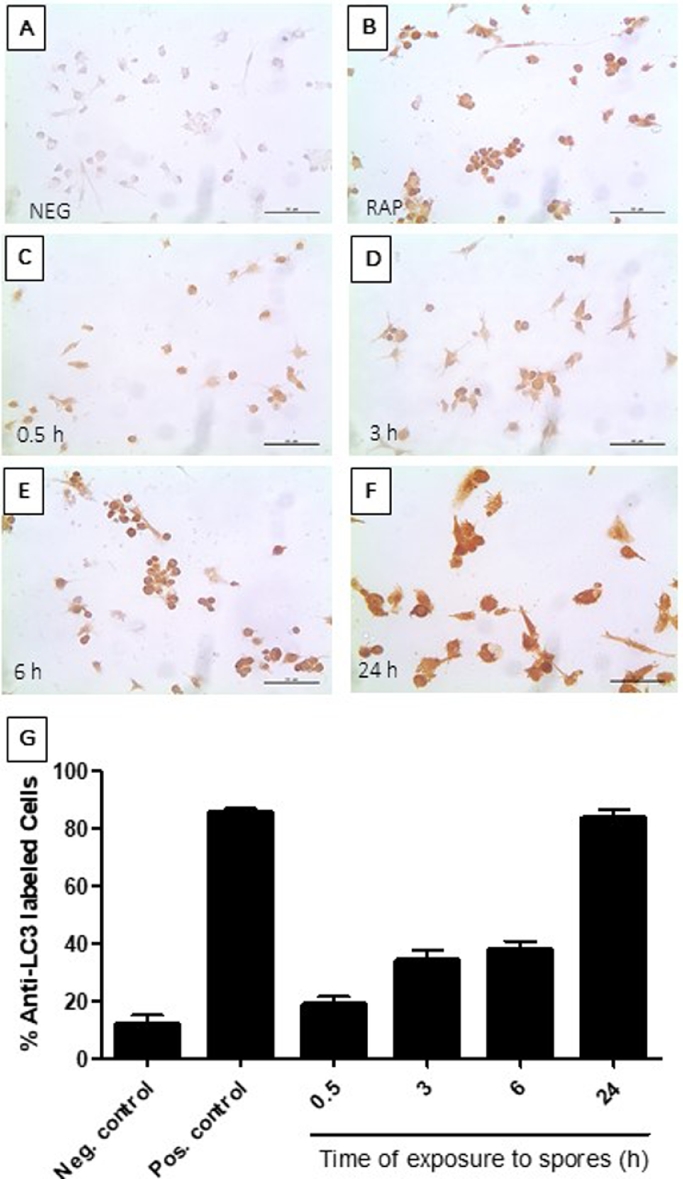
Detection of autophagy marker LC3 in human macrophages before and after exposure to T. stromaticum spores. (C-F) Immunoperoxidase using anti-LC3 antibody. Cells (A) not exposed to spores; (B) exposed to 100 ng rapamycin; exposed to spores for (C) 30 min; (D) 3h; (E) 6 h (F) 24h. (G) Percentage of cells stained with antibody anti-LC3. Data represent the mean ± SD of 3 independent experiments.

We then investigated whether *T. stromaticum* spores induce autophagy also in human macrophages, as previously seen in J774 cells. Here, we employed immunocytochemistry method and optical microscopy, using an antibody anti-LC3B, an autophagosome membrane marker that plays a central role in the autophagosome membrane structure (Martinet et al., 2013), searching for stained vacuoles. When in contact with *T. stromaticum* spores, human macrophages, displayed cumulative amounts of AVs with increasing exposure times with *T. stromaticum* spores, as verified by the presence of LC3B-stained cells (Fig. 4C-4F), comparable with cells exposed to rapamycin as a positive control (Fig. 4B). In 24 h, almost 90% of the cells were stained with anti-LC3B as cells treated with rapamycin (Fig. 4G).

**Fig. 5:**
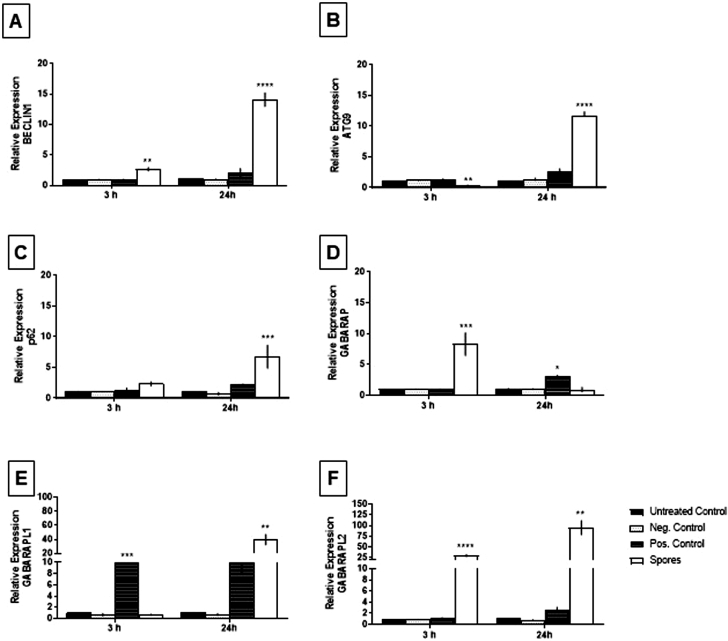
Relative expression of autophagy related genes. Human macrophages obtained from human peripheral blood were cultivated without treatment (untreated control), exposed to beads (Negative control), exposed rampamicin (Positive control) and exposed to T. stromaticum spores for 3h and 24h and the gene expression analysis was evaluated by quantitative PCR. (A) BECLIN 1, (B) ATG9, (C) p62, (D) GABARAP, (E) GABARAP L1, (F) GABARAP L2 gene expression values. The relative expression was calculated using the 2^-∆∆Ct^ methodology and values were normalized with the GAPDH gene. Data is represented by mean ± SD of three independent experiments by ANOVA

Next, we examined whether the induction of autophagy by *T. stromaticum* spores correlates with the modulation of molecular mechanisms of autophagy induction controlled by ATGs. *BECLIN1, GABARAP, GABARAPL1, GABARAPL2, ATG9* and *p62*, fundamental genes related to this process, were analyzed (Klionsky et al., 2021).All genes analyzed in human macrophage exposed to *T. stromaticum* spores were upregulated, as compared to unexposed group with distinct kinetics (Fig. 5A-F). Upregulation of *Beclin 1*, involved in the initiation stage of autophagy, was already observed at 3 h after treatment of the cells with *T. stromaticum* spores, and further increasing at 24 h to around 15-fold (Fig. 5A). *ATG9* and *p62* increased 24h after exposure to spores (Fig. 5B, C). As to the *ATG8* gene family (*GABARAP, GABARAP*L1, *GABARAP*L2), involved in the transduction of LC3B, and associated with the maturation of autophagosome in eukaryotic cells, both *GABARAP* and *GABARAPL2* increased during the first 3h. The levels of *GABARAP* returned to basal levels after 3h, whereas *GABARAPL2* continued augmenting until 24 h , reaching around 100-fold basal expression (Fig. 5D, F). *GABARAPL1* transcripts had also a substantial increase of around 40-fold the basal levels, which, unlike the other members of this family, occurred only 24h after exposure to spores (Fig. 5E). Upregulation of these genes upon contact and ingestion of spores by macrophages is particular for the spores, since ingestion of inert particles, such as the cytometer beads used, does not alter their expression. Rapamycin, a standard inducer of autophagy, used here as a positive control, increases the expression of some of the genes, particularly after 24 h. It is possible that the rapamycin is not altering all genes due to different required concentrations or time frame of maximal expression.

### 3.4 ***T. stromaticum* spores modulate the production ROS and downregulate *NLRP3* expression*, IL-1***β **and *IL-18* in human macrophages**

**Fig. 6:**
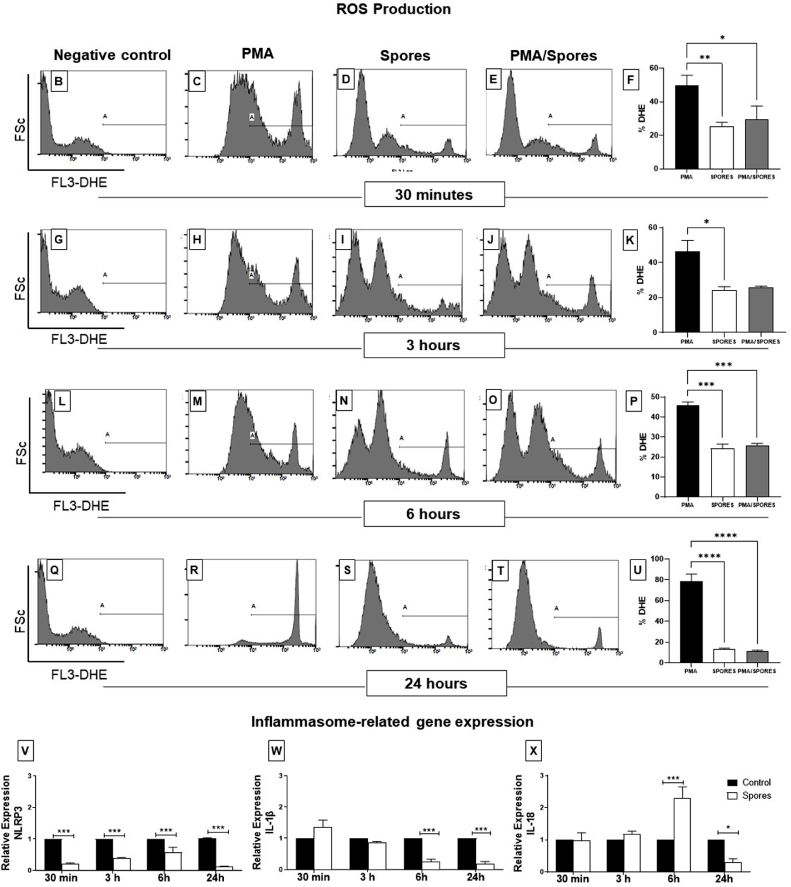
Detection of ROS production and transcriptional levels of inflammasome related genes in human macrophages after *T. stromaticum* exposure*.* Human macrophages exposed to PMA, *T. stromaticum* spores and spores + PMA were incubated with the Dihydroethidium (DHE), a probe that enters in the cellular cytoplasm and once oxidized emits fluorescence. Fluorescence was detected by flow cytometry after 30min (B-E), 3h, (G-J) 6h (L-O) or 24h (Q-T) of stimuli, summarized on panels F, K, P and U. The gate A represents the percentage of DHE-positive cells during acquisition of 20,000 events by Flow Cytometry. Data is shown as mean + standard deviation; ANOVA followed by Bonferroni post-test. (*) p < 0.05, (**) p < 0.01 and (***) p < 0.001. Data is representative of three different experiments. Expression of the genes *NLRP3* (V), *IL-1β* (W) and *IL-18* (X) in human macrophages exposed or not to *T. stromaticum* spores during 30min, 3h, 6h and 24h were obtained by qPCR using three independent experiments. The relative expression was calculated using the 2^-∆∆Ct^ methodology and values were normalized with the GAPDH gene. Data is represented by mean + SD by ANOVA with Bonferroni post-test. Bars represent mean + SE (n=5 replicates/group). (*) p≤0.05 and (***) p≤0.001 compared to control group.

Following the observation that spores of *T. stromaticum* induce autophagic vacuoles and affect the expression of autophagy genes in human macrophage, we sought to determine whether spores were able to alter cell ability to produce ROS and cytokines related to the activation of NLRP3 inflammasome. For ROS detection, we have used DHE, a probe that enters in the cellular cytoplasm and, once oxidized, emits fluorescence. PMA is a classical ROS inducer, used here to test the ability of the spores to inhibit ROS production. We defined the fluorescent-positive A gate, using untreated cell (Fig. 6 B, G, L and Q). Our results demonstrated that cells treated with spores promoted around 50% reduction in PMA-induced ROS production beginning at 30 min of treatment (Figs 6 C, E and F; H, J, and K; M, O and P) and almost 90% at 24 h (Fig. 6 R, T and U), levels equivalent to cells treated with only spores, which initially induced the production of ROS in 20% of the cells. Only the peaks inside the A gate are the probe-fluorescent cells. Other alterations outside the A gate, are inside the negative fluorescence intensity, defined in untreated control (Fig. 6 B, G, L and Q), and are not significant for the conclusions drawn. These above results are summarized in figure 6 F, K, P e U for the fluorescence of the indicated A gate.

Autophagy can control inflammation through the negative regulation of key inflammatory mediators, such as NLPR3 components, and consequently cytokines such as IL-18 and IL-1β. This is accomplished by the removal of endogenous inflammasome activators, such as ROS-producing damaged mitochondria, and inflammasome components and cytokines (Biasizzo and Kopitar-Jerala, 2020). We thus sought to determine the expression of NLRP3, IL-18 and IL-1β genes to evaluated whether *T. stromaticum* spores was also able to disrupt inflammasome response in human macrophages. We observed that spores induce a strong downregulation of *NLRP3* gene expression, already at 30 min, which lasts and increase in 24 h (Fig. 6V), followed by a downregulation of the expression of *IL-1β* (from 6h and 24h Fig. 6W) and of *IL-18* (at 24h - Fig. 6X), the latter preceded by a transient upregulation.

### 3.5 *T. stromaticum* spores alter the expression of *CLEC7A, TLR2, TLR4, IL-10, IL-12* and miR-146 and mir-155 in human macrophages

**Fig. 7:**
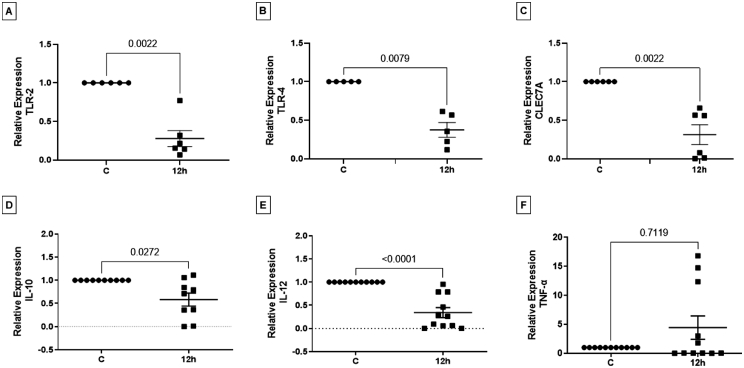
Relative levels of PRRs and cytokines in human macrophages exposed to *T. stromaticum* spores. Human macrophages cultured in the presence of *T. stromaticum* spores had the levels of (A) *TLR2*, (B) *TLR4*, (C) *CLEC7A*, (D*) IL10*, (E) *IL12*, and (F) *TNFα* expression evaluated after 12 hours of exposure by qPCR. Results represent the mean of two independent assays together, with confidence interval of 95%. Asterisks indicate significant differences relative to the control (p < 0.05), by Tukey test analysis.

Following the observation that spores induce autophagic vacuoles and and ATGs, as well as modulate ROS production and inflammasome components in human macrophages, we sought to investigate whether the spores also affected other functional characteristics of the cell. We then evaluated the expression of some PRRs, namely TLR2, TLR4 and CLEC7A, as well as the cytokines IL-10, IL-12, and TNFα. Our results showed a reduction in the expression of TLR2, TLR4 and CLEC7A (dectin-1), as compared to unexposed control cells (Fig. 7A-C), as well as a downregulation in the expression of IL-10, IL-12 (Figs 7D and E). No effect was observed regarding the production of TNF-α (Figure 7F).

**Fig. 8:**
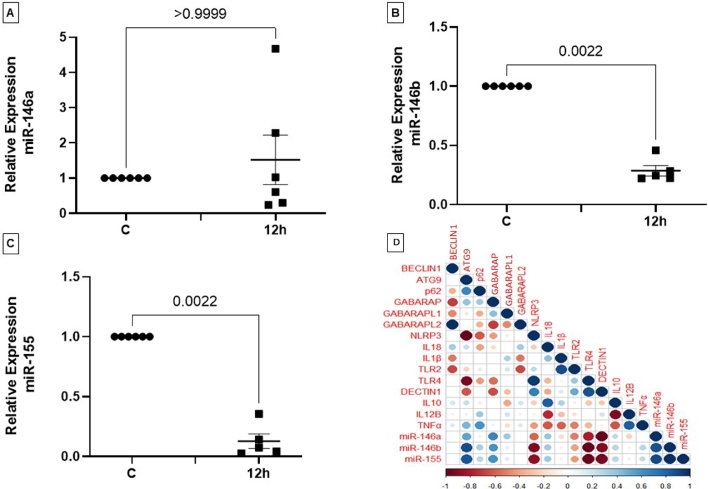
Relative levels of miRNAs and PRRs in macrophages exposed to T. stromaticum spores. Macrophages cultured in the presence of T. stromaticum spores had the levels of (A) miR-146a, (B) miR-146b, (C) miR155, expression evaluated after 12 hours of exposure, by qPCR. Results represent the mean of three independent assays in duplicate together with confidence interval of 95%., by Tukey test analysis. (D) Correlation matrix between levels of all genes analyzed in this study and miRNAs. Circles and corresponding sizes represent the significance level. Colors represent the directionality of the correlation (blue - positive correlations; red - negative correlations).

Since the microRNAs 146a/b and 155 regulate the phagocyte response to distinct pathogens, and are widely involved in pro-inflammatory transcription programs, we also investigated whether *T. stromaticum* spores affected the expression of these microRNAs. After 12 hours of exposure to *T. stromaticum* spores, the expression of miR-146b (Fig. 8B) and of miR-155 (Fig. 8C) decreased by around 3-fold and 10-fold, respectively. Expression of miR-146a was not affected by the spores (Fig. 8A).

Considering that these innate immunity pathways addressed in our study could be associated with miRNAs, we correlated the expression levels of miRNAs and autophagy, PRRs and cytokine genes. The correlation matrix presented in Fig. 8D summarizes these results. Expression of microRNAs showed positive correlation with the genes *ATG9, p62, GABARAP, IL18* and *IL10* and a negative correlation with the genes *NLRP3, TLR2, TLR4* and *CLEC7A*). Furthermore, we observed that the autophagy genes are correlated.

## 4 Discussion

The scarcity of studies related to the effect of the biofungicide Tricovab®, formulated with the fungus *T. stromaticum*, on the immune system is remarkable. In PubMed web site (https://www.ncbi.nlm.nih.gov/pubmed), as accessed on 10 June 2022, of the 3,927 hits containing the keyword *Trichoderma* in the title of the article, only 7 (0.18 %) contain the words *Trichoderma* and *stromaticum*, none containing the word Tricovab®. This is worrisome, since this biofungicide has been used in Brazil and other countries for controlling the witches’ broom disease of cocoa trees (del Carmen et al., 2021, Medeiros et al., 2010; ), people are exposed to it and it effects on human health is very poorly investigated. Previous studies from our laboratory have demonstrated many suppressive effects in mice immune system and human neutrophils, particularly in its innate branch (Alves-filho et al., 2011; dos Santos et al., 2017; Konstantinovas et al., 2017; Oliveira-Mendonça et al., 2022). So far, no studies were made using a human macrophage model. Here, we decided to investigate the impact of *T. stromaticum* spores in the innate immune response focusing on the on human macrophages.

In observing the interaction of *T. stromaticum* spores with mice J774 macrophages we observed signs of autophagy, such as AV and MF, as well as LD (Fig. 2), which prompt us to investigate other signs of autophagy in these cells as well as in human macrophages exposed to the fungus. The presence of of MDC-positive autophagosomes in J774 macrophages corroborated the morphological data (Fig. 2). Autophagy after contact/ingestion with *T. stromaticum* spores was also induced in human macrophages, as shown by the increased the expression of LC3b (Fig. 4), a microtubule-associated light chain protein of the ATG8 family related to the elongation phase of the autophagic process (Dikic and Elazar, 2018) and of genes related to autophagy, such as *ATG9, p62, BECLIN1* and *GABARAP* isoforms (Fig. 5). Upregulation of these genes implies that they may be actively involved during the autophagosome maturation upon human macrophage contact with the spores. Considering the kinetics of the expression of the ATGs seen in this study, we observed that *BECLIN*1, a key regulator in autophagy, participates in the initial process, being expressed within the first 3 hours of spore contact, and is positively correlated with the *GABARAPL2* when compared in the correlation matrix (Fig. 8). This indicates the relationship of both genes in the formation of autophagosomal membranes (Hu et al., 2021). In addition, other genes of the ATG8 family (*GABARAP and GABARAPL1*) showed a positive correlation with each other, although expressed with different kinetics. GABARAPL1 activates the ULK1 complex while GABARAP recruits ULK1 to initiate phagophore formation, inducing vesicle growth, being also important in the process of phagophore closure (Jacquet et al., 2021). Expression of *GABARAP* within the first 3 hours of exposure to spores indicates its relationship with the formation of the autophagosome, whereas the overexpression of *GABARAPL1* only after 24 hours of spore treatment, indicates a function for this gene in the final step autophagy, for instance the fusion of autophagosome with lysosomes (Jacquet et al., 2021). With regard to ATG9, a regulator phagophore formation, essential for autophagosome biogenesis and maturation, being a critical regulator number of autophagosome (Hu et al., 2020), we observed an overexpression only after 24 hours of exposure to spores, demonstrating its importance in a later stages of autophagy. A positive correlation of the *ATG9* gene with *p62*, also overexpressed only after 24 hours of spore treatment was detected (Fig. 8). p62 is not related to the number of autophagic vacuoles, but acts as a substrate through autophagic degradation, being essential for the aggregation ubiquitinated proteins in the proteasome (Liu et al., 2016).

Autophagy is essential for innate immunity and is involved in mediating a variety of innate immune pathways (Cui et al., 2019). It is an important mechanism to maintain cellular, tissue and organism homeostasis being mediated by ATGs, which allows the degradation and recycling of components in the cytosol (Levine and Kroemer, 2019). Hence, it is probable that the induction of autophagy by *T. stromaticum* spores in mice and human macrophages have an impact in immune regulation and host defense.

Pathogens can modulate autophagy mechanisms as a means of survival to avoid action by other effectors of the immune system. It has been described that *Listeria monocytogenes* promotes mitophagy, a selective autophagy, reducing cellular ROS levels and, at the same time, increasing bacterial survival (Zhang et al., 2019). By promoting selective autophagy, they attenuate inflammation, so that they block or activate steps within the autophagy pathway to optimize its intracellular permanence (Sanchez-Garrido and Shenoy, 2020). Filomeni et al., 2015 have reported that the induction of autophagy may be due to oxidative stress ()(). In fact, we show here that *T. stromaticum* spores may induce the production of ROS, which could be involved in the induction of autophagy (Fig. 6). On the other hand, they also strongly inhibited a PMA-induced production of ROS, suggesting that this important antimicrobial mechanism, which is also involved in immune signaling and induction of inflammasome activation (Herb and Schramm, 2021), may be also impaired in humans that have inhaled *T. stromaticum* spores. Under normal physiological conditions, cells maintain a dynamic equilibrium between the production and clearance of ROS. An imbalance in ROS production can promote infection or other disorders (Mannan et al., 2021). ROS production can be associated to membrane damage and fungal enzymes are able to cause membrane disruption (El-Gendi et al., 2022) and *Trichoderma reesei* liberate cellulases-containing extracellular vesicles that present molecules involved in lysosomal/endosomal transport regulation (De Paula et al., 2019).

Autophagy caused by *T. stromaticum* spores were also accompanied by reduction in the expression of genes involved in inflammation, such as NLRP3, IL-1β and IL-18. This is possible due to ROS inhibition (Hennig et al., 2018). However, is not still clear how ROS integrates all the signals to inflammasome assembly. NLRP3 inflammasomes activation is an essential mechanism for fighting intracellular pathogens. This protein complex responds to a wide range of stimuli leading to the production of pro-inflammatory cytokines IL-1β and IL-18 (Angosto-Bazarra et al., 2021). Autophagic machinery also has a role in unconventional secretion of IL-1β and thus regulates inflammatory response. Conversely, NLRP3 inflammasome activation regulates autophagosome formation through several different mechanisms. Crosstalk between inflammasomes and autophagy is necessary for balance between required host defense inflammatory response and prevention of excessive inflammation. In addition to NLR inhibition, *T. stromaticum* spores could also downregulate the expression of TLRs and dectin receptor, as well as of IL-10 and IL-12, but not of TNF-α, corroborating that the spores immunomodulate the immune response. Increased number of lipid droplets in the cytoplasm of macrophage that ingested the fungal spores may be relevant for subsequent immune response as these structures display enzymes involved in the regulation of immune response and inflammation (Pereira-Dutra and Bozza, 2021) and the NLRP3 inflammasome activation is related to modulated lipid droplets formation (Dong et al., 2020).

MiR-146 and miR-155 are known by their ability to differentially contribute to endotoxin-inflammatory responses (Das Gupta et al., 2014). The first maintains a sub-inflammatory immune response or decrease the human leukocyte activities, while the second has pro-inflammatory characteristics (Nahid et al., 2009; Schmidt et al., 2009). Since miR-155 has an inflammatory effect while miR-146 has an anti-inflammatory effect (Testa et al., 2017; Zhao et al., 2016). Furthermore, despite the common mechanism of NF-κB transcriptional regulation for miR-146 and miR-155, they differently control the TLR4 signaling pathways, which plays important role in phagocytes for fungi infection recognition (Curtale et al., 2013; Griss et al., 2016). The fact that we showed that the expression of miR-146b and miR-155 decreased, after treatment with *T. stromaticum* spores. TLR4 and IL-1R signaling pathways that lead to inflammasome activation are targeted by microRNAs such as miR-155 (Boxberger et al., 2019). Although some studies indicate that miR-155 promotes autophagy (Wan et al., 2019; Zhang et al., 2017), in this study we verified the formation of autophagic vacuoles with low miR-155 regulation, suggesting that autophagy-related genes can regulate the inflammatory components of cytokines and microRNAs. miR-155 is well described as pro-inflammatory, has numerous target genes and has been reported to inhibit autophagy (Holla et al., 2014). According to our correlation matrix results, miR-155 is positively correlated with autophagy-related genes, while it is negatively correlated with PRRs. This agrees with the literature, since in our results there was a reduction in miR-155 and PRRs and induction of autophagy, characterizing the spores of the fungus *T. stromaticum* with an immunosuppressive effect.

A few effects of *T. stromaticum* on murine immune cells, shown in previous work (Alves-Filho et al., dos Santos et al., 2017), such as the downregulation of dectin-1, TLR2 and TLR4, was also confirmed in the present work for a more relevant model of human macrophages. Importantly, besides showing similar results to the murine model, it aggregates many more newly described altered functionalities of human cells when in contact with *T. stromaticum* spores. In describing these phenotypes, we hope to learn which other macrophage abilities are affected by this biocontrol agent.

Exposure to *T. stromaticum* spores may impact human immune responses in two important clinical aspects: the first regards to the safety of their use in agriculture as a biological control tool. Individuals in rural areas could be regularly exposed to large doses of spores that could potentially act as inhibitors of inflammatory responses and become susceptible to infection. Once the *T. stromaticum* spores are the basis the Tricovab®, biofungicide sprayed a in cocoa crops for controlling *Moniliophithora perniciosa*, its safety, particularly its effect on human immune response, must be better elucidated. The second, the immunosuppressive effect of these spores may be used therapeutically in diseases characterized by exacerbated immune responses, such as autoimmune diseases, transplantation, or hypersensitivity. Since the spores a composed of multiple molecules, they could be explored regarding their different components as to obtain immunosuppressive or anti-inflammatory immunopharmaceuticals.

## Author Contributions

**“**LSOM, MMS, AGdS and JLS conceived and designed the experiments; MMS, LSOM, CMK, AGdS, DOdA, NASS, KPL, JFS, GRA and MGC performed the experiments; LSOM, MMS, AGdS, JLS, IBA, and MAVS analyzed the data; JLS, CMK, ISJC and IBA contributed reagents/materials/analysis tools; JLS, SOD, DGA, EAM, MFH and LSOM analyzed the results and wrote the paper.”

## Declaration of Competing Interest

The authors declare no conflict of interest.
